# The Subjunctive as a Model of Grammatical Complexity: An Integrative Review of Issues Based on Combined Evidence from Mental Chronometry and Neurosciences

**DOI:** 10.3390/brainsci13060974

**Published:** 2023-06-20

**Authors:** Daniel Grégoire Grevisse, Marzena Watorek, Frédéric Isel

**Affiliations:** 1 Centre National Recherches Scientifiques, Unité Mixte de Recherche 7023 Formal Structures of Language, University Paris 8, 93200 Saint-Denis, France; 2 Centre National Recherches Scientifiques, Unité Mixte de Recherche 7114 Models, Dynamics, Corpora, University Paris Nanterre, 92000 Nanterre, France

**Keywords:** subjunctive complexity, EEG, fMRI, psycholinguistics, neurocognition of language

## Abstract

The acquisition of a second language requires the construction or reconstruction of linguistic knowledge about the new language system. Learners of a second language have to acquire the linguistic structures of the second language by constructing or reassessing their own knowledge in the light of the new one. Some of these new linguistic structures may be more or less complex to process and/or difficult to acquire. In this review, we focus on an example of linguistic complexity in French, namely, the subjunctive. Through a discussion of some selected studies on the second language acquisition of the French subjunctive, our purpose is to argue that these findings, considered from a psycholinguistic perspective, could be fruitful for further research employing neuroscience techniques, such as electroencephalography or neuroimaging in order to better understand the neurocognitive processing of this complex structure both in French native speakers and in learners of French. Hence, we aim to contribute to exploring the question of linguistic transfer in the field of second language acquisition, the typological distance/relation between L1–L2, the syntactic acquisition of complex structures in adult second language learners, and the potential contributions of electroencephalography and functional magnetic resonance imaging to the processing of the subjunctive, selected as an example of linguistic complexity that has not yet received much attention.

## 1. Introduction

While certain linguistic structures may prove difficult for native speakers to acquire, their characteristics (syntactic, phonological, etc.) may make them even more difficult for second language learners (henceforth L2). The processing of these structures is assumed to have a higher cognitive cost. When acquiring an L2, learners have to construct and reconstruct their linguistic knowledge. That is, some of the morphological categories of an L2 may be difficult for learners to acquire because they involve retrieving in memory the grammatical rules that govern the use of the morphological categories. This is the case, for example, for the acquisition of the French partitive articles, both in positive and in negative sentences, by Italian learners of French L2, see [1].

Conversely, other structures that are easier to acquire may prove complex because syntactic procedures, such as dependencies, have to be respected (e.g., the agreement of the French past participle in compound tenses) [2]. Lastly, some grammatical features, such as the grammatical tenses of Romance languages (Italian, French, Spanish, etc.), may be complex to process and difficult to acquire for learners whose L1 expresses tenses differently (e.g., Japanese and Chinese), using different grammatical categories (adverbs, adverbial locations, etc.). This is the case, for example, with the difference, in French, between the *imparfait* (*Il fermait la porte* … ‘He was closing the door …’) and the *passé composé* (*Il a fermé la porte* … ‘He closed the door …’), which are difficult and complex for Chinese learners of French L2 to acquire and process [3,4].

Here, we review psycholinguistic L2 studies that have jointly or separately examined *complexity* in processing and *difficulty* in acquisition of L2. Our goal is to better understand why certain structures of an L2 are difficult to acquire and complex to process, but this is the originality of the perspective taken by this review by relating these structures to those of the L1. We adopt here a typological point of view by discussing the typological relationship between L1 and L2 for the linguistic phenomena studied. To do so, we have taken as an example of linguistic complexity the subjunctive in French. We will see why the French subjunctive can be considered complex, both from a linguistic and an acquisitional point of view, by analyzing its formal and functional grammatical characteristics. In order to understand the cognitive processes that are involved during the processing of complex syntactic structures by L2 learners, we discuss two neurocognitive models that might help to account for the processing of linguistic complexity in L2 acquisition, namely, the *Shallow Structures Hypothesis* [5,6,7,8] and the *Declarative/Procedural Model* [9,10,11,12,13]. These two neurocognitive models will be useful to answer the following research questions: (1) does *complexity*, observed at different levels of linguistic analysis, involve, qualitatively, and quantitatively, the same neurocognitive processing mechanisms in native speakers and in L2 learners? (2) what role does the level of proficiency in the L2 perform in the functioning of these mechanisms? (3) how does proficiency in L2 impact the acquisition and production of linguistic complexity in the L2? and (4) in what way does the typological distance/relation between L1–L2, for a specific syntactic L2 phenomenon, influence the processing of linguistically complex structures and their difficulty of acquisition? As already stated, we will consider these questions in relation to an example of linguistic complexity, i.e., the French subjunctive.

The article is structured as follows. First, we briefly discuss the difference between *complexity* of processing and *difficulty* acquiring specific linguistic structures in studies of L1 and L2 development and in studies of neurocognitive processing. As an illustration of linguistic complexity, we focus on the present subjunctive mood in French. This will be an opportunity to understand why the French present subjunctive can be considered psycholinguistically complex to process and cognitively difficult to acquire. Second, in order to understand the processing of this mood in French, we will discuss selected studies on the acquisition of the present subjective in French L1, then in French L2. We will present selected neurocognitive studies investigating how the subjunctive in English and German is processed by native speakers. Then, we discuss how these studies and the neuroscience techniques employed, i.e., electroencephalography (EEG) and functional magnetic resonance imaging (fMRI), allow us to better understand how the complexity inherent to the present subjunctive is processed both by French natives and French L2 learners. Finally, we discuss two neurocognitive models of L2 acquisition: the *Shallow Structures Hypothesis—*SSH [5,6,7,8] and the *Declarative*/*Procedural Model*—D/P [9,10,11,12,13], and we will propose some perspectives on the processing of the French subjunctive in L2 based on the typological distance/relation. Our main aim here is to explain why the French present subjunctive can be considered as an example of linguistic *complexity* and why it may represent a *difficulty* of acquisition in French L2.

## 2. *Difficulty* of Acquiring and *Complexity* of Processing of an L2

In general, scholars agree that the difficulty of acquiring new linguistic structures in L2 is cognitively associated with a higher demand for computing resources, such as working memory [14,15,16], which means that learners make cognitive efforts to understand, store, retrieve, and to build their own linguistic structures of an L2. Many psycholinguistic studies (see [17] for a review) have pointed out the extent to which some linguistic structures in L2 are difficult to acquire and complex to process for L2 learners and also difficult to use in their productions, both oral and written. Nevertheless, to our knowledge, none of these studies has answered the question of “why” some structures are difficult to acquire and complex to process. Researchers (see [18]) claimed that *difficulty* is related to the cognitive cost, in terms of working memory, during the processing of L2 linguistic structures by learners and that it is also related to learner characteristics, such as motivation, aptitude, L1 background, L2 proficiency, development stage in L2, among others. Thus, as underlined by Bulté and Housen ([17] (p. 23)), the *difficulty* of acquiring a language feature is to some extent subjective or learner-dependent.

DeKeyser [14] discussed some factors that may influence the degree of difficulty in acquiring L2 structures. He proposed that five factors interact with L2 acquisition. These are the age of acquisition (AoA), individual cognitive differences, the characteristics of the L2, the influence of the L1, and the learning context. He considered that a factor that determines the difficulty of acquisition of an L2 is the transparency between form-meaning relationships. Accordingly, it is hard for the learner to understand the relation that exists between the form itself and the meaning it may have based on the context. Furthermore, DeKeyser [14] argues that even without taking into account the form for expressing the meaning, what makes it difficult in L2 acquisition is the meaning itself because it could be novel, abstract, or both for the learners. For example, some studies [19,20,21,22,23,24,25,26,27,28,29,30,31] reported that articles, grammatical gender, verbal tenses, and aspects are hard to acquire regardless of the type of instruction (formal or informal) that the learners receive. As shown by this research, complexity, and difficulty are not always related. In fact, difficulty and complexity, is modulated and determined by several factors which are not necessarily related [14].

As language features influence *difficulty*, this means that the notion of *difficulty* is not determined only by subjective factors. It is sufficiently clear that objective factors, which are more language-dependent, have an impact on the degree to which an L2 language feature is perceived as *difficult*. These language-dependent factors are assumed to determine the level of complexity of a language. Among them, we can consider lexical factors (collocations, and lexemes), morphological (inflection, derivation), syntactic (sentence, clause, and phrase), and phonological ones (segmental, suprasegmental) [18]. Different definitions and typologies of *complexity* have been proposed [17,32,33,34,35,36,37,38,39] but there appears to be a general consensus that *complexity* is determined by the relations that exist between the elements of a system and their hierarchical organizations at different levels of the system in our case at different levels of language structure. In syntax, morphosyntactic complexity is defined by the number of connections a sentence is made of and the dependencies this structure has. The notion of *complexity* has been a matter of interest for many scholars from different linguistic backgrounds. In fact, *complexity* is a well-known property in grammar, where we usually distinguish the simple from the complex sentence by arguing that complex sentences are made of different types of dependencies. In linguistics, *complexity* became a subject of interest only recently but research has exploded in different fields, ranging from the formalist perspective, where most research concerned syntax and phonology [40,41,42,43,44,45,46,47,48], to the functionalist approach, where different dimensions of *complexity* were explored, from different points of view, such as diachronic, synchronic, typologic, and L1 and L2 acquisitional [32,33,35,36,37,49,50,51]. In terms of acquisition, psycholinguistic research usually uses different measures to account for linguistic complexity in task-based studies for different linguistic domains (syntactic, lexical, and morphological), such as mean length of T-unit, utterance, T-unit (see [17]) in both L1 and L2 acquisition and development. These measures allow scholars to report the degree of linguistic complexity produced by children of an L1 and learners of an L2 and to determine the linguistic domain that is concerned.

Interestingly, the linguistic description of grammatical complexity seems to be reflected in processing at the neurocognitive level. In fact, from the neurocognitive point of view, concerning the issue of syntactic complexity at the heart of this review, Kaan and Swaab [52] (see also [53]) showed that the processing of syntactically correct sentences could modulate the amplitude of a frontal electroencephalographic component called the P600, i.e., a positive deflection peaking around 600 milliseconds after the critical event under investigation, as a function of their syntactic complexity. Therefore, they argued that the frontal P600 related to complexity has to be separated from the well-known posterior P600 that was previously associated with repair and revision in syntactically anomalous sentences ([24,52,53,54,55,56,57,58,59] among others). Both Friederici, Hahne, and Saddy [53], and Kaan and Swaab [52] showed that the frontally distributed positivity (frontal P600) is related to ambiguity resolution and/or to an increase in grammatical complexity while a central-posterior positivity (P600) is related to syntactic repair, revision, and unification of sentences. Usually, in L1 processing, a biphasic event-related potential (ERP) pattern LAN/P600 was found in many studies, including morphosyntactic processing violations and complexities, such as gender, number, and subject-verb violations, but also in garden path sentences, canonical vs. non-canonical sentence order, preferred vs. non-preferred sentences (see [60,61,62,63,64]. This biphasic pattern is suggested to reflect first morphosyntactic violations and complexity detection for the LAN effect (LAN (Left anterior negativity) is a negative deflection in the anterior part of the left hemisphere occurring between 300–500 ms and reflecting morphosyntactic violation detections (see [53,54,55,56,57,63,64,65])), and second, revision/repair for the P600 ERP component. In neurocognitive studies on L2 acquisition, researchers typically look for the same biphasic LAN/P600 pattern found in native speakers to determine the degree of native-like processing in bilinguals. In particular, they study the qualitative and quantitative variation of amplitude, peak latency, and surface topography of the ERP thought to reflect the processing of a linguistic phenomenon in L2 learners in comparison with native speakers. However, different factors influence native-like processing, such as proficiency (or efficiency) level in L2, AoA of an L2, dominance of L1 and L2, time spent in an L2-speaking country, type of L2 instruction (formal vs. informal), training duration of L2, L1–L2 similarities (see [66], for further details), and the typological relation between L1 and L2 for the structure concerned. Scholars are far from finding this ERP biphasic pattern, which is LAN/P600, systematically in L2 learners’ processing (see [67] for a discussion, also [61]). This lack of consensus is attributed to the fact that LAN is considered to be linked to a more automatic, implicit grammar processing, whereas P600 is a less automatic and more controlled ERP pattern. In this paper, we will assume that the biphasic ERP pattern LAN/P600 displays native-like processing in late bilinguals as shown in some studies [68,69,70]; see [66] for a detailed review).

## 3. The French Subjunctive as an Example of Linguistic Complexity and Difficulty

It is generally agreed that all languages have the subjunctive but that it can be expressed differently (lexico-semantically, morphosyntactically) depending on the language’s typology (e.g., Chinese and Japanese vs. Italian, French, and Spanish). In this section, we will discuss the French subjunctive. If we assume with Bulté and Housen ([17] (p. 24)) that *complexity* is defined as “the number of discrete components that a language feature or a language system consists of, and as the number of connections between the different components”, we can claim that the subjunctive, in French, constitutes an example of linguistic complexity, as this mood denotes abstract concepts (e.g., marking irrealis events and states) and has a syntactic distribution that is largely limited to subordinate clauses [71]. More specifically, we can consider that the French subjunctive might be *complex* and that it might represent a *difficulty*, for L1 and for L2 acquirers, for three reasons. First, from a morphological point of view, the subjunctive has a complex conjugation, particularly for the verbs of the third group, ending in *-re/oir,* such as *tendre* ‘to stretch’, and *recevoir* ‘to receive’. In fact, the subjunctive in French is not formally marked in all its forms. Additionally, this is the second reason the subjunctive can be considered complex. When we say “unmarked” we refer to the fact that orally, the subjunctive cannot be distinguished from the indicative, as in the sentence *Je préfère que tu vois* [vwa] *toi-même* ‘I prefer that you see for yourself’ (indicative) vs. *Je veux que tu voies* [vwa] *toi-même* ‘I want you to see for yourself’ (subjunctive). In speech, only the verbs of the third group, ending in *-re/oir*, are phonetically marked and can therefore be differentiated from the indicative (*Je préfère que tu le reçois* [ʁəswa] ‘I prefer you to receive him’ _indicative_ vs. *Je veux que tu le reçoive* [ʁəswav] ‘I want you to receive him’ _subjunctive_. Lachet (2013), [72] proposed to account for the complexity of subjunctive morphology and phonetics by considering three forms of this mood: (1) unmarked: [mãje] > *il mange* ‘he eats’ (indicative and subjunctive); (2) marked but not-subjunctive specifically: [tjɛn] > *qu’ il tienne* ‘that he keeps’ (subjunctive) vs. [tj$\tilde{ɛ}$] > *il tient* ‘he keeps’ (indicative) but also [tjɛn] > *ils tiennent* ‘they keep’ (indicative); (3) subjunctive specifically marked: [fas] > qu’*il fasse* (subjunctive) vs. [fɛ] (il) fait (indicative). Third, it is assumed that the subjunctive is the mood of subordination: in embedded clauses, the subjunctive appears following a triggering element (lexical: subordinating conjunctions such as *avant que* ‘before that’, or grammatical: verbs, such as *vouloir que* ‘wish that’) present in the main clause [73,74]. According to Grevisse and Goosse [73], the subjunctive is the ideal mood of subordinate clauses. The authors point out that it is used for expressing wishes, suggestions, desires, uncertainty, fear, supposition, and prohibition. It is introduced by subordinating conjunctions, such as *afin que* ‘so that’, *pour que* ‘so that, *à moins que* ‘unless’, *bien que* ‘although’, *malgré que* ‘despite the fact that’, *de crainte que* ‘for fear that/lest’, *de manière que* ‘in such a way that’, *de façon* (*à ce*) *que* ‘so that’, etc. In contemporary French, the most frequently used subjunctive tenses are the present and the past, while the *imparfait* (e.g., *Je souhaitais qu’il vînt* ‘I wished he would come’) and *plus-que-parfait* (e.g., *J’aurais souhaité qu’il fût venu* ‘I wish he had come’) are employed only in the literature and in formal registers (administrative documents). Furthermore, the subjunctive can also be used in main clauses when they are in the imperative, the injunctive, the exclamatory, or optative forms ([73] (pp. 1102–1105), [74] (pp. 564–566)). The indicative and the subjunctive can also alternate in some syntactic structures. Interestingly, researchers [75] showed that some particularities in the main clause (e.g., the subject), the negative form of the sentence, in contexts of presupposition, and adverbial negation facilitate this modal alternation (indicative/subjunctive).

In conclusion of this section, if we go back to our definition of *complexity*, it is easy to understand why the subjunctive, given its formal and functional grammar and morphosyntactic characteristics, can be considered an example of *complexity of processing* and *difficulty* of acquisition in both French L1 and L2. For French natives, the complexity of the subjunctive and the difficulty of acquiring it are automatically computed at the cognitive level. This might not be the case for French L2 learners, however, who maybe need first to understand the role of the subjunctive, to distinguish its modalities from indicative modalities, and then learn how to use it. Considering these particularities, it is likely that this verbal mode may be difficult for learners of French to acquire and to produce and also complex to process.

Before reviewing the research on French L2 acquisition, we will discuss the matter of the French subjunctive in native speakers, specifically in children.

### 3.1. Acquisition of the Subjunctive by French Native Speakers

There are only a few studies that have accounted for the acquisition of the subjunctive in French L1. In all of them, the subjunctive was not the main target of the research but just one topic among others, suggesting that this mood is still understudied in the field of acquisition and neurocognition. Still, linguistic research on L1 acquisition of the French subjunctive showed that this mood is acquired at different stages of language development in children. Parisse, Pontox, and Morgenstern [76], using a spoken corpus of French (project CoLaJE) composed of oral productions of children (six children, all French natives, aged 0–7, from higher social backgrounds) reported, for example, that moods, such as the conditional and the subjunctive are produced at the age of 4, while more frequent moods, such as the present and the past indicative, are already observed in most of the two-year-old children [76]. Interestingly, the authors also found some occurrences of the subjunctive in some of the two-year-old children. Based on this evidence, Parisse, Pontox, and Morgenstern [76] argue that at the age of 2 some verbal tenses can be evidenced, such as the present and the past, but also some moods, such as the imperative, conditional, and subjunctive ([76] (p. 154)). According to their research, at the age of 4, children already have good adult-like mastery of the subjunctive.

These findings were not confirmed, however, by Bassano [77], who used an oral corpus of 2 French native children, aged 2–4 and 1–4 years, recorded longitudinally, and the cross-sectional corpora of the so-called “TRL” database composed of recordings of children at 20, 30, 39, and 48 months of age (each age group consists of 20 children, with the exception of recordings of children at 20, 30, 39, and 48 months, each age group comprises 20 children, half of whom were followed longitudinally). He found that it was only at the age of 5 that French children started to use the subjunctive in conversation and that it accounted for only 6% of verbal forms at the age of 7. Bassano argued that the acquisition and development of verb tenses and moods are influenced by prosodic properties (such as the syllable structure and the rhythmic group) of linguistic structures and only at a later age by lexical factors. In another study, Bassano, Maillochon, Klapfer, and Dressler [78] found that their participant, a French child aged 1;3 (who was longitudinally recorded up to the age of 3), produced the subjunctive with high-frequency verbs, such as *faire* (‘to do’), *abîmer* (‘to damage’). Taken together, these production data in French children converge in suggesting that the subjunctive is acquired and produced by French natives in the early stages of language development. It has been shown that the stage of emergence is around two years old, and the age of full acquisition and adult-like use is around the age of 7. These findings lead to two major conclusions. First, they show that the subjunctive is not as difficult as it is thought to be since it does not present a problem of acquisition for French natives. Second, despite statements by some linguists about the death of the subjunctive in spoken French (see [79,80,81,82]), this mood seems to be still alive and to be used sufficiently often in a speech to enable children to acquire it early in life, as attested by the above-mentioned studies.

Findings in French native acquisition studies are in accordance with other production studies that explored the acquisition of the subjunctive in other languages, such as Spanish. For example, Dracos, Requena, and Miller [83] analyzed the mood selection of 66 Spanish-speaking children (age range 4;02–10;03) and 13 adults in a sentence-completion task (selection of verbal mood). Their results indicate that children master subjunctive use, at an adult-like level, by age 6/7. The authors suggest that the selection mode is context-specific and is suggestive of the processing complexity. Furthermore, it seems that semantics performs an important role in the acquisition of the subjunctive, as demonstrated by Pérez-Leroux [84], who investigated the impact of cognitive maturation (sufficient resources thanks to the complete maturation of relevant brain structures) in children who had to understand false beliefs. Twenty-two Spanish speakers aged 3–5 and 6–11 took part in an elicited production study. Pérez-Leroux concluded that even if children acquire and produce subjunctive morphology early, they need to understand and differentiate true beliefs from false ones (The moon is made of rock vs. The moon is made of cheese) in order to use it in relative clauses in the subjunctive mood where a semantic representation is needed to decode the sentence. Finally, Avrutin and Wexler [85] used a truth-value judgment task, which consisted in asking children to make a bipolar judgment related to a previous context (“yes” or “no” the pronoun referring to the previous context), with 8 Russian-speaking children aged 4 to 5 years old. The aim was to analyze the impact of syntax and discourse-related constraints on the interpretation of pronouns in subjunctive clauses. The authors showed that when only syntactic knowledge is required for processing pronouns in subjunctive sentences (to identify that the pronoun in the subjunctive clause is not coindexed with the matrix subject), children have an adult-like use of the syntactic rules of the subjunctive. However, things are different in sentences where the correct interpretation of the pronouns is restricted to discourse-related elements and the interaction of syntactic rules: in these cases, children make more mistakes of interpretation.

All these results from production studies show that children of different typological L1 (French, Russian, and Spanish) acquire and have a good command of the subjunctive early in their developmental stages of L1 acquisition. They have an adult-like command of the subjunctive at the age of 4 or 7, depending on their L1, but when pragmatic knowledge is needed (to differentiate true beliefs from false ones, for example), cognitive maturation is required. Nevertheless, one might claim that children may produce subjunctive forms as an unanalyzed formulaic sequence. To resolve this question, Clahsen and Felser [5] proposed the continuity hypothesis for children’s L1 processing. The authors suggest that children use the same mechanism as adult speakers without any change over time. They suggest, however, that differences in performance between children and adults could be accounted for by limited working memory and/or limited access to the mental lexicon for children. These interpretative hypotheses are supported by some studies [86,87,88,89,90]; see [5,6,7,8] for more details evidencing that, for children, the problem is to retrieve the lexical-semantic properties of lexical units and to reformulate sentences. Importantly, empirical evidence suggests that children have difficulties in applying pragmatic information (such as how to use language or how to adapt it to different social contexts) during phrase parsing (=processing) and that they have difficulties when sentence ambiguity resolution is needed, specifically during online processing (e.g., garden path sentences). Furthermore, as suggested by Ullman [10,11], even if some linguistic forms of L1 may be stored in the mental lexicon, given the fact that associative lexical memory is able to generalize new structures from memorized forms, the lexicalization process may lead to productivity. This point of view leads to refuting the idea that the memorization and the production of new linguistic forms is an unanalyzed cognitive process. Rather, both processes include both lexical forms and morphosyntactically complex structures [9,91].

In conclusion, it does not seem plausible to consider that the forms of the subjunctive produced by children are unanalyzed structures. This view is in accordance with the principle of the elicited imitation task, which consists of repeating, as accurately as possible, what the participant has just heard. The idea is that if the participant does not have the structure in his grammar, he will never be able to reproduce it [92]. In the same way, a child will never (re)produce a subjunctive if it is not part of their grammar. Thus, we can consider the subjective forms produced by children and attested by empirical studies as fully analyzed structures.

In the following section, we address the question from the perspective of L2 acquisition.

### 3.2. Acquisition of the Subjunctive in French L2: A Review of Selected Production Studies

In this section, we first present some psycholinguistic studies that investigated the acquisition of the subjunctive by French L2 learners from different L1 backgrounds, namely, Italian, Spanish, Finnish, Turkish, Russian, and English. This cross-linguistic approach will allow us to consider both the diversity of difficulties encountered in learning the subjunctive and the role performed by the learner’s L1.

Among the most well-known studies on the acquisition of the French subjunctive is the work by Bartning (Ref. [93]; Swedish) and Bartning and Schlyter [94]. Their research was based on two linguistic corpora: the first one comprised different linguistic tasks (see below) conducted in France, including oral interviews with 99 Swedish learners of French L2 aged between 19 and 34, who had studied French for a period between zero and nine years, and 20 native speakers of French. Tasks were spread over a period ranging from two semesters to two years, divided into two parts consisting of a 15 min interview and 20 min narratives of a comic strip and a silent film. The second corpus consisted of recordings of oral productions of 20 Swedish adults aged between 19 and 70 (8 of whom were living in France, 7 were studying French at university in Sweden, and 5 were just beginning to learn French). For these 20 participants, tasks were spread over a period of 2 to 12 months and included an interview, a story-tale, and a translation.

Following the analysis of the occurrences of the subjunctive in the two corpora, Bartning and Schlyter [94] determined an acquisitional pathway of the subjunctive divided into six stages. They proposed six stages of acquisition of the subjunctive as follows: {1} no contexts with the subjunctive; {2} rare occurrences of *il faut que* (‘it is necessary that’) and *je veux que* (‘I want that’) + V (infinitive or past participle co-occurrence or *il faut que* (‘it is necessary that’) + V (infinitive or past participle) and *il faut que* (‘it is necessary that’) + neutralized form/indicative (e.g., *il faut que j’écris* ‘It is necessary that I write’); {3} emergence of contexts of *il faut* (‘it is necessary’) *+ INF* and *il faut P* (‘it is necessary P’) (with infinitives and indicatives); {4} emergence of subjunctive in mandatory contexts in correct (*il faut que j’aille* ‘It is necessary that I go’) and incorrect forms (*pour que le couple va ‘*for the couple to go’), coexistence of two structures *il faut que* (‘it is necessary that’) *+ INF* and *il faut que* (‘it is necessary that’) *+ SUBJ*; {5} good command of the structure *il faut que* (‘it is necessary that’) *+ SUBJ* and with different conjunctions, late emergence of contexts with negative verbs without the subjunctive (e.g., *je ne pense pas que je peux* … ‘I don’t think that I can …’) [85] in all contexts the subjunctive is used correctly. In addition to this acquisitional pathway, Bartning and Schlyter ([94] (p. 293)) proposed to group the six stages into clusters that define characteristic profiles in order to be able to place learners in developmental stages. The six developmental stages they proposed are: {1}—initial; {2}—post initial; {3}—intermediate; {4}—low advanced; {5}—intermediate-advanced; {6}—high advanced. Each of these developmental stages is characterized by the appearance of different grammatical features in the acquisitional pathway. According to Bartning and Schlyter [94], while the subjunctive context may be found at the intermediate stage, it is only completely mastered at different advanced stages (depending on the linguistic typology distance/relation).

It is worth noting that before Bartning and Schlyter [94], Brum de Paula [95] proposed 4 stages of acquisition of verbal moods for the Brazilian participants in his study, who were L2 French learners. In his study, Brum De Paula [95] states that the subjunctive is acquired in the final stage (the fourth one), and that it is used with high-frequency verbs such as *pouvoir* ‘can’, *vouloir* ‘to want’, *faire* ‘to do’. This method of the acquisitional pathway is very useful for understanding how the subjunctive emerges and develops over time in learners of French L2. The weakness of the method, however, is that it does not inform us about why the acquisition of the subjunctive occurs only at stage 6 (or 4 in the case of de Paula) in advanced learners and not before. Some researchers argued that the typological distance/relation between the L1 and the L2 could perform an important role. In fact, Kellerman [96] claimed that “the strategy of ‘transfer’ of native language (NL) items into target language (TL) expressions is considered here to be an active learner strategy dependent on the learner’s notion of ‘distance’ between the NL and the TL. Some NL items will be more liable to transfer than others to the extent that they are believed to be less native language-specific”. This claim opens the way to the notion of psychotypology. Tokowicz and MacWhinney [79] confirmed this hypothesis by showing that the participants in their study (EEG) were more sensitive (i.e., they recognized both grammatical and ungrammatical sentences) in L2 to sentences that were similar in L1 and L2 but they were unable to detect violations in the constructions that differed in L1 and L2. Evidence from both production and neurocognitive studies shows that learners are more sensitive to linguistic structures similar in L1 and L2, whereas they are not sensitive to those that are different in L1 and L2, regardless of the level of proficiency in the L2.

This was demonstrated in a psycholinguistic production study by Badalamenti (2013) on Italian learners of French L2. Two different studies were carried out to analyze the emergence of different grammatical structures in French L2; the first one was longitudinal, and the second was cross-sectional. Her corpus was based on 75 Italian university students of French L2, aged between 18 to 54. They were divided into two groups: (1) group A: students whose objective was to be teachers of French L2 and (2) group B: students in political sciences (see Badalamenti [97], for more details). Results showed that the subjunctive stabilized only at an advanced stage (i.e., stage {4}), as shown by Bartning and Schlyter [94], ([97] (p. 269)). Badalamenti speculates that only the completive clauses followed by an infinitive are acquired early during second language acquisition. She also claims that multi-propositional clauses involving the subjunctive and conditional moods are observed late. Linguistic proximity between French and Italian seems to perform an important role in French L2 acquisition of structures that are similar in both languages but not for the acquisition of the subjunctive, even if some functional aspects (e.g., verbs expressing modalities, such as command, wish, suggestions, desires, etc.) are similar in Italian and in French. The close typology relation thus appears to influence the acquisition of this mood for Italian learners of French L2.

We will now discuss the role of the typological relation in the acquisition of the French subjunctive in more detail by reviewing selected psycholinguistic studies investigating Finnish, Turkish, Russian, and Anglophone learners of French. In Finnish, Paloheimo [98] evaluated the acquisition of Tense, Aspect, Mood (TAM) by Finnish learners of French from a database of written texts (TAITO). This archive is composed of 40 texts written by first-year university students specializing in French philology. Paloheimo found that the verbal forms of the subjunctive are not always transparent, i.e., they cannot be distinguished from the indicative (e.g., *Je mange* [ʒəm$\tilde{a}$ʒ]_indicative_ ‘I eat’ vs. *Il faut que je mange* (ilfokəʒəm$\tilde{a}$ʒ)_subjunctive_ ‘I have to eat’) and that students, therefore, confound the subjunctive with the present tense of the indicative (*Je mange une pomme* ‘I eat an apple’ vs. *Il faut que je mange une pomme* ‘I must eat an apple’). For the students who used the subjunctive, it appeared to be employed with conjunctions that trigger it in subordinate clauses when the subject in the main and the subordinate clause is the same. Some rare contexts with the imperfect subjunctive were also found. She suggests placing those students who employed the subjunctive in the low advanced stage of acquisition proposed by Bartning and Schlyter [94]. In fact, Carlo ([99] (p. 163)) observed that the speed with which verbal modality marking is acquired in French L2 depends on the typological proximity of the learner’s mother tongue with French. Furthermore, Paloheimo indicates that the subjunctive is used simultaneously with the conditional. This finding is similar to that of Brum De Paula [95], who observed that Portuguese learners of French L2 used the subjunctive simultaneously with the *plus-que-parfait* tense of the indicative and the conditional. It can be inferred from this that the L1 may be a facilitator of the acquisition of the subjunctive for Portuguese learners because the subjunctive exists in Portuguese. Unlike Portuguese and Italian, the Finnish verbal system does not have an equivalent for the subjunctive. To express it, Finnish speakers must use all the semantic components equivalent to the original clause in French (Ingo, 1990, [100]). These semantic components can be expressed by the indicative, conditional, or imperative as in this example where the French subjunctive is translated in Finnish by a conditional: Fr. *Puissiez vous*_subjucntive_ *être à tous les diables* (Molière), Finnish. *kunpa hiisi teidät korjaisi*_conditional_ *kaikkineen päivineen* (Leino) ‘Could you be to all devils’. It is intriguing to note that, as discussed by Paloheimo [98], Finnish advanced learners of French used the conditional in their productions before using the subjunctive but after having used the future tense of the indicative. Paloheimo concludes that this may be explained by the influence of the L1, Finnish. In fact, the future tense does not exist in Finnish, whereas the conditional is present in the formal grammatical construction of Finnish. Its presence in Finnish thus facilitates the acquisition of the French conditional but not of the French subjunctive.

The issue of transfer in second language acquisition had already been discussed by Lado [101], who proposed two types of transfer: positive, when the linguistic structures in L1 and L2 are similar, and negative, when the linguistic structures of the two languages differ. Topaloğlu [102] found evidence in production supporting the transfer hypothesis for the acquisition of the subjunctive by Turkish learners of French L2. The subjunctive exists in Turkish, but it differs from French both in morphological/formal structure (in Turkish there are twenty-two different verbal forms) and in function (in French there are eighteen domains of usage while in Turkish there are only four; see [103] (pp. 336–337) for more details). Topaloğlu [102] hypothesized that what creates difficulties for students in the acquisition of the French subjunctive is the morphological and functional differences between the L1 and L2. Participants attested that it was complicated for them to find an equivalent of the French subjunctive in Turkish and that this slowed down the learning process. The typological distance/relation with Turkish learners’ L1 makes it difficult for them to understand the use and functions of the subjunctive in French. Additionally, participants declared that it was difficult for them to differentiate between the subjunctive and the indicative when these modes were formally identical (e.g., *Je cuisine* ‘I cook vs. *Il faut que je cuisine* ‘I have to cook’). To overcome this problem, learners rely on their L1 even if the transfer is not positive (in Lado’s terms).

Another parameter to consider when studying the process of the acquisition of time, aspect, and mood of an L2 is the frequency of occurrence of the linguistic tokens in the learners’ input (see Ellis [104] for a review). It seems that the frequency of occurrence in the L2 under acquisition influences the acquisition and production of verbal morphology. In an elicitation task with Russian learners of French that consisted in eliciting the conjugated forms of third, fourth, and sixth persons of the present indicative, the third person of the present subjunctive, and the third person of the simple future, past participle and infinitive of high-frequency verbs (*faire* ‘to do’, *prendre* ‘to take’ and *devoir* ‘to have to’) and low-frequency verbs (*boire* ‘to drink’, *craindre* ‘to be afraid of’ and *traduire* ‘to translate’), Sergeeva and Chevrot [105] investigated the production of conjugated forms of the French verb paradigm, with a particular focus on the choice of verbal bases. The authors aimed to define the role performed by the frequency of tokens and the presence of base forms in the verbal paradigm. Tasks were administered to 30 Russian learners of French L2 at the university level. Participants were distributed in two levels: (1) half of them were in their second year of history and journalism studies, and (2) half of them were in their fourth year, specializing in the literature. They were divided into two groups: low level for those studying journalism (N1) and high level for those studying literature (N2). Findings suggest that, for the N2 group, frequent verbs are better used than infrequent ones; this difference was not found in group N1. The correlation between the frequency of bases and forms of verbs was higher in the N2 group than in the N1 one. Sergeeva and Chevrot [105] concluded that the selection of verbal bases is compelled to affect the frequency of tokens even in cases where the selection of a base variant is fundamental for expressing the person, the tense, and the mood. This is the case for the subjunctive, for which the third PERS is more frequent than other persons, so it is acquired earlier than other persons.

Other researchers studied the influence of socio-pragmatic and semantic competence in the acquisition and use of the French subjunctive as L2. Howard [106] examined the expression of modality through the subjunctive mood based on the oral productions of 20 English-speaking learners of French L2 at the advanced (6) level (see Table 1). Participants were divided into three groups: group 1 composed of students who had finished their second year of university and who had never spent a long time in a French-speaking country, group 2 composed of students who had spent one year in a French-speaking country, and group 3 composed of students who had finished their bachelor’s degree, majoring in French. What emerged from his qualitative analysis is that only a few forms in the subjunctive were indexed, as shown in the table below adapted from Howard ([106] (p. 6)).

Howard observed that even if students created contexts where the subjunctive should have been used, they did not use it and when they used it, only *falloir* (‘it is necessary to …’) triggered the use of the subjunctive in subordinate clauses. Howard concluded that students, even at an advanced level, whether they had lived in a French-speaking country or not, seldom produce subjunctive forms but that they produce more contexts where the subjunctive could be used. His conclusions (see also Howard [107], for a comparative study on naturalistic and instructed L2 exposure to French) are in line with those of the study by Isabelli and Nishida [108] on the acquisition of the Spanish subjunctive, which also concluded on the poverty of subjunctive production after studying in a French country.

Concerning the period spent abroad and the acquisition process, McManus and Mitchell [109] investigated, on the one hand, the production of the subjunctive in speech and writing and, on the other hand, whether different triggers were used with the subjunctive in writing and oral productions, and lastly, whether the subjunctive employed with different triggers changed over time in French L2 English-speaking university learners’ productions (speech and writing). Their participants were 29 students, all native speakers of English and learners of French, who spent nine months in France. A control group of participants was composed of ten native speakers of French. Their data were based on oral and written tasks and a grammaticality judgment test (GJt). The authors also investigated whether students produced the subjunctive before their departure and the nature of the forms and triggers that they produced. They analyzed the development of students’ acquisition during their stay in France. This helped them to state the benefits of studying abroad during L2 learning. Their results show that students produced few subjunctive forms before leaving and that their production did not increase much during their stay abroad. Their results also showed that “it is more frequently used in writing than in speech” [109]. On the nature of different triggers, results showed that students were more capable of identifying affirmative forms (e.g., *Il veut que* ‘He wants that …’) than subordinating conjunctions or negative clauses forms (e.g., *Je ne crois pas que* … ‘I don’t believe that…’). The authors concluded that “it appears that affirmative subjunctive triggers represent a key source of development, with most change evident for lower proficiency learners” [109]. Two important points are worth highlighting. First, their results are in line with those of Bartning [94,110,111], suggesting that triggers, such as *il faut que* ‘it is necessary that’, *il souhaite que* ‘He wants that’, *il est important que* ‘It is important that’, *Il doute que* ‘He doubts that’, etc., are important cues for students to better use the French subjunctive. Second, as suggested by McManus and Mitchell [109], affirmative triggers (see above) are used by high-proficiency learners. This means that these triggers are acquired and processed by the learners. These findings are in accordance with those of Badalamenti [97] concerning Italian speakers of French L2. It seems that proficiency in French L2 performs a crucial role in acquiring, understanding, and producing the subjunctive. It is immaterial how proficiency is increased, by time spent in countries where French is spoken, by studying at home, or by exchanging with native French speakers; what is important is to gain proficiency. It is unquestionable that some of these pedagogical practices are better than others; studies have shown that spending time in a French-speaking country may be better than studying by oneself.

Proficiency was investigated in the study by Mitchell, Ventura, and McManus [112]. Twenty-nine English-speaking learners of French L2 participated in their inquiry. All participants were majoring in both French and Spanish. Additionally, all participants spent one year in France, and two of them were compound bilinguals (they were early bilinguals of both French and English). In order to examine the production of the subjunctive, participants took part in two tests: speech production and writing. The speech production part consisted of interviews on general questions. The researchers inventoried all the triggers and contexts likely to induce the use of the subjunctive. Their findings indicate that high-proficiency participants produced the subjunctive more frequently than the lower-proficiency ones. Furthermore, they discovered that even if participants used different syntactic triggers, they were not used in the same proportion because “just five of them account for 64% of all SUBJ contexts in L2 speech (*falloir que* ‘to have to’, *vouloir que* ‘to want to’, *ne pas penser que* ‘don’t think that’, *avant que* ‘before …’, and *pour que* ‘to …’) (Mitchell, Ventura, and McManus [112] (p. 88)). Overall, it appeared that accuracy intensified during the informants’ stay in France. The writing task involved essay writing. The results for this task indicated that the subjunctive was much more frequently found in writing than in speech. The authors also noted an increasing ability and greater linguistic variety of the subjunctive and structures in the subjunctive mood in writing than in speech. Lastly, Mitchell and coauthors [112] found that in oral production, participants used only verb triggers, while conjunction triggers were found only in writing.

In a recent study, Dudley and Slabakova [113] used eye tracking (factors inspected: eye fixations, convergence, and saccades) to investigate online sensitivity to the subjunctive in French L2. Forty-five English-speaking learners of French (34 females, *mean age* = 14.42, *SD* = 1.12 years, first exposure to French at 9.47 years) in their second and final year of an undergraduate degree or their one-year postgraduate degree in modern and foreign languages took part in the study. Participants performed three tasks: an eye-tracking reading task, an acceptability judgment task, and a lexical test. The research questions sought to determine whether sensitivity was modulated by L1–L2 differences in verbal mood featural formal configurations and whether the level of proficiency performed a role in the detection of errors involving subjunctive use. Results from the acceptability judgment task showed that participants had knowledge of the French subjunctive but were unable to apply their knowledge when reading the sentences [113]. The eye-tracking test failed to demonstrate any evidence regarding the L2 learners’ sensitivity to mood mismatches (violation paradigm). While native speakers of French showed sensitivity to the mood mismatches (regressions back to the critical region for the final part of the sentences, long reading times for the subjunctive, a significant regression-out probability effect for directive verbs), no significant effect of mood-modality mismatches were found among L2 learners of French.

Taken together, all the studies presented above state that the subjunctive is a great model of grammatical complexity and that it is difficult to acquire. From the above-mentioned studies, the following generalizations can be made to account for the difficulty of acquiring this complexity in French. It seems that proficiency is an important factor that influences the acquisition of the subjunctive. The studies showed that only intermediate-advanced learners produce it correctly. Furthermore, it was underlined that the linguistic typological relation between L1–L2 performs an important role. As evidenced by Badalamenti [97], Tokowicz, and MacWhinney [103], for example, when the subjunctive mood exists in the learners’ L1 and is used as in the L2, it may be simpler to acquire. However, when the subjunctive is expressed differently in the learners’ L1 or when some structures differ in L1 and L2, it may be more difficult to acquire and complex to process.

The following section presents the case of processing the subjunctive by native German and English speakers in order to account for how future research should proceed for applying it to French.

## 4. Neurocognitive Studies Investigating the Neurophysiological Activity in Processing the Complexity of the Subjunctive

As already stated, to our knowledge, no studies to date have investigated the acquisition of the French subjunctive by French learners adopting neuroscience techniques, such as EEG or fMRI. However, there is evidence from research using EEG and fMRI of processing clauses containing the subjunctive in German and English by native German and English speakers. We discuss what these studies show us about the cognitive mechanisms underlying the processing of the subjunctive and how findings from EEG and fMRI are useful for developing research in French. We start by presenting the case of processing the subjunctive in German (Section 4.1), then the case of the English subjunctive (Section 4.2).

### 4.1. The Counterfactual Subjunctive in German: fMRI and EEG Evidence

Counterfactuals refer to events that could have happened but did not. They also denote facts that contradict the real world (e.g., If the moon were made of cheese). To study the processing of counterfactuals, Kulakova and colleagues [114] used the fMRI technique in German. As German has no conditional, irrealis and hypothetical values are expressed by employing the so-called type II subjunctive (type I is restricted to formal language). However, some conditional forms can be formed with the indicative when the information is accessible or real. In their study, Kulakova and colleagues [114] compared structures containing a subjunctive that contradicted the explicit facts of the real world—hence the notion of counterfactuality—(*Wenn der Motor heute an wäre, … würde er dann Treibstoff verbrauchen?* ‘If the engine were on today, … would it then consume fuel?’) to structures containing the indicative and expressing hypotheticality (*Wenn der Motor gestern an war, hat er dann Treibstoff verbraucht?* ‘If the engine was on yesterday, did it use fuel?’), which did not contradict the facts represented by the real world and thus reflected an almost expected hypothetical assumption and where the assumption lost its unreal character. They tested 21 native German speakers using both written and spoken German sentences which were composed of 18 Counterfactual (CF) (*Der Motor ist heute aus. Wenn der Motor heute an wäre, … würde er dann Treibstoff verbrauchen?* The engine is switched off today. If the engine were switched on today, … would it burn fuel?) and 18 hypothetical (HYP) (*Der Motor ist heute aus. Wenn der Motor gestern an war, … hat er dann Treibstoff verbraucht?* The engine is switched off today. If the motor was switched on yesterday, … did it burn fuel?) sentences. Participants were instructed to read the visually presented sentences carefully (or to listen to them in the auditory condition) and to decide about the consistency between the antecedent and consequent events. They were encouraged to rely on their common sense and world knowledge instead of logical or probabilistic considerations.

The results showed activation in the right occipital cortex (cuneus) and the right basal ganglia (caudate nucleus) during the processing of counterfactual sentences for both the visual and the auditory presentation, which excluded a visual processing artifact. Generally, the occipital cortex is associated with mental imagery [115], i.e., the construction of imaginary events and the integration effect of the upcoming information (see [116]), and as the basal ganglia in the right hemisphere are activated during truth evaluation, factually false sentences and integration [54,117]. Consequently, Kulakova and colleagues [114] interpreted these results by proposing that while processing counterfactual sentences, the individual has to keep in mind both real events and events that might have happened but did not. Additionally, with this kind of sentence, one has to inhibit irrealis events in order to consider only events that are real. They also proposed that activation of the basal ganglia could be associated with a linguistic mode of processing, in their case, with the differentiation between the indicative and the subjunctive (see [118] for syntactic complexity processing). However, as they found that this activation was right-lateralized, they interpreted it as an effect of semantic integration and interpretation of the counterfactual sentences.

In another study implying grammaticality judgment and probe detection tasks, Kulakova and coauthors [119] analyzed the ERPs of seventeen participants, all native speakers of German. The material consisted of conditional clauses in the subjunctive (counterfactual) (*Wenn die Würfel gezinkt wären*_subjunctive_*, dann wäre das Spiel fair/unfair.* ‘If the dice had been_subjunctive_ rigged, then the game would have been fair/unfair.’) and in the indicative (*Wenn die Würfel gezinkt waren*_indicative_*, dann war das Spiel fair/unfair*. ‘If the dice were rigged_indicative_, then the game was fair/unfair.’) ([119] (p. 3)). The sentences were presented visually, one word at a time. As claimed by the authors (p. 3), in the counterfactual, the auxiliary “wären” (critical word underlined) “marks subjunctive mood and allows the reader to infer that although it is supposed that the dice were rigged (suppositional meaning), they in fact were not (factual meaning). In the indicative condition, “waren” introduces the suppositional reading only, allowing no inferences about the factual state of affairs”.

ERPs were recorded at the point of mood disambiguation in the antecedent and were compared between the conditional clauses in the subjunctive and in the indicative. Results showed a transient negative deflection in frontal regions for the subjunctive compared to the indicative mood in a time window of 450–600 ms. The authors discussed their results with respect to working memory requirements for rule application and increased referential processing demands for the representation of counterfactuals’ dual meaning. The authors suggested that when counterfactually is involved, then the dual meaning is processed from the first moment at the earliest point where counterfactually is marked by subjunctive mood.

### 4.2. The Counterfactual Subjunctive in English: EEG Evidence

In their study, Kulakova and Nieuwald [120] used the EEG/ERP for understanding whether pragmatic knowledge of the world could influence the processing of counterfactual sentences and, more importantly, how this knowledge may condition the processes involved in the processing of counterfactual sentences. For this purpose, they investigated six conditions: truth conditionals (If sweets were made of sugar …), false counterfactual (If words were made of sugar …), true hypothetical (If sweets are made of sugar), false hypothetical (If words are made of sugar …), true declaratives (As sweets are made of sugar …), and false declaratives (As words are made of sugar …). Thirty students (19 women, mean age = 22 years, and 11 native English speakers) took part in the study. The authors concluded that the N400 marker is implicated during the processing of the following case: “words that are consistent with factual knowledge incur a semantic processing cost, as reflected in larger N400 amplitude, in counterfactual antecedents compared to hypothetical antecedents” ([120] (p. 814)). Pragmatic knowledge seems to be important during the neuronal processing of counterfactuality. According to the researchers, counterfactual clauses in the subjunctive are integrated as soon as processed, and they reduce expectations based on factual knowledge.

This EEG and fMRI evidence of the processing of the subjunctive in English and German L1 could enable future research to better understand the neural underpinning of how the subjunctive is processed in native speakers of French. On the one hand, as underlined by Kulakova and colleagues [114], since the basal ganglia, in the right hemisphere are activated during the processing of allomorphy, it could be relevant to test this hypothesis in French L1 processing of the subjunctive. In fact, the present subjunctive in French has allomorphy for the 2nd and 3rd groups (ending in *ir* and *oir/re*), which allows differentiation with the present indicative. On the other hand, Kulakova and Nieuwald [120] showed that pragmatic information and semantic processing are implicated during the processing of counterfactuals in English. One might wonder if semantic information is considered when processing the subjunctive in French and to what degree semantics and syntax interact in order to build a relevant interpretation of the sentence.

In conclusion, while it is interesting to use neuroscience techniques in order to know how, when, and where the processing of the complexity of the subjunctive takes place, some researchers accounted for the difficulty of acquiring and producing and also of the complexity of processing some L2 structures by proposing different neurocognitive models. In order to better understand the difficulty of acquiring certain complex linguistic structures in L2, we will discuss two neurocognitive models that may help us to explain why L2 complex linguistic structures are difficult to acquire and produce. These two neurocognitive models are the Shallow Structure Hypothesis [5] and the Declarative/Procedural Model [11,12,13].

## 5. Neurocognitive Models of L2 Acquisition: The *Shallow Structure Hypothesis* and the *Declarative/Procedural Model*

We will focus on two models of L2 acquisition of syntactic complex structures, the *Shallow Structures Hypothesis* (SSH) [5,6,7,8] and the *Declarative/Procedural Model* [9,10,11,12]. We will first discuss the Shallow hypothesis, then the D/P model.

Based on research in processing morphosyntactic structures by L2 learners, especially relative-clause ambiguities and filler-gap dependencies (see [8]), and the difficulties these studies revealed for L2 learners to acquire some complex L2 morphosyntactic structures, Clahsen and Felser [5,6,7] suggested considering that language processing in adult L2 learners, especially when it comes to sentence processing, is rather different from the processes employed by L1 children. In fact, the authors consider that the neurocognitive mechanism of acquisition by L1 children has the same neurocognitive correlates as those of the adults, suggesting that there is continuity over time and that neurocognitive processes do not change at a mature age. In contrast, they suggest adult L2 learners’ sentence processing is driven more by lexical-semantic, pragmatic information, and neuronal mechanisms, and syntactic information is less implicated. Therefore, they consider that the processing of L2 complex morphosyntactic structures in adult L2 learners is “shallower and less detailed than those of native speakers” ([5] (p. 3)). In other words, this model of real-time sentence processing in adult L2 assumes that even if the adults are highly proficient in L2, there is a tendency to process some complex syntactic structures of the L2 (e.g., relative clauses, embedded clauses, anaphora, case marking, and *wh-*questions) by semantic, pragmatic, discourse, surface-level cues, and mechanisms. It is important to note that, as underlined by the authors, the SSH does not assume that L2 learners rely only on superficial structure-building information, but it suggests that in the case of processing L2 complex syntactic structures, L2 learners “compute grammatical representations that lack complex hierarchical structure” ([6] (p. 111)). This means that in some cases, especially when the syntactic structures of the L2 are relatively complex/difficult or they do not exist in the learners’ L1, to overcome the problem of processing them, learners may rely on semantic, pragmatic, and discourse cues. Since this hypothesis was first proposed, many studies have pursued the objective of clearly understanding which L2 syntactic and morphologic structures are superficially processed by adult L2 learners and why (see [121] for a discussion).

An alternative model is the Declarative/Procedural model (D/P) proposed by Ullman [9,10,11,12]. His hypothesis is based on declarative and procedural memories. The declarative memory system supports the use of knowledge concerning facts and events, the learning processes, and the representation. Procedural memory, in contrast, subserves the implicit (nonconscious) learning of new elements and controls skills and habits requiring sequences. Thus, declarative memory is thought to be more explicit and rely on the mental lexicon, while procedural memory is implicit and implicated in mental grammar.

According to Ullman [12], given the facility for adult L2 learners to rely on declarative memory, they use declarative memory to process structures that should be processed by the mechanisms of procedural memory, such as syntactic structure building, even when these structures are subserved by the procedural system in their L1. Thus, memorization is a widely diffused process that adult L2 learners use and prefer for processing complex structures in an L2. However, given the storage limitations of the declarative system, according to Ullman, it cannot supply all the procedures ensured by the procedural/grammatical system, and this may explain the deficit in attaining native-like processing in all aspects of the L2 grammar. Ullman suggests that with experience in L2, adult L2 learners should become more efficient in L2 syntactic processing, relying more on the procedural/grammatical system, and proposes some factors that should be considered in order to attain nativelike neurocognitive processes, such as the type of syntactic structure to be acquired, the nature of exposure to the L2, the individual characteristics of the learners, and intrinsic procedural learning skills.

These two models may help us to account for the difficulty in acquiring and the complexity of processing the French subjunctive in French L2. In fact, on the one hand, it may be useful to consider that when structures in the subjunctive mood in French L2 are processed by learners from languages where the subjunctive exists (e.g., Italian) but these structures differ in learners’ L1, it could be difficult to assess and stabilize the differences. On the other hand, for learners where the subjunctive is expressed differently in their L1 from French (e.g., Chinese), the processing of the French subjunctive may be more lexico-semantically than morphosyntactically driven until a very high level of proficiency is attained based on learner’s experience with French, nature of exposure to French, instruction, AoA of French, or immersion in a French-speaking country.

## 6. Conclusions: New Perspectives for Further Inquiry into French Subjunctive Acquisition

This review of questions aimed to disentangle the different factors accounting for the *complexity* of the French subjunctive. Our goal here was to review combined linguistic, psycholinguistic, and neurocognitive studies that investigated the acquisition of the subjunctive in French L2 by different learners in comparison to French native speakers. The originality of this review is to present the contribution of mental chronometry studies, including factors such as typological relation between L1 and L2, proficiency, and syntactic acquisition of an L2, to the study of how syntactic complexity, such as the subjunctive in French is processed, and how these results might help us to better understand the processing of linguistic complexities by using different neuroscience techniques (EEG, fMRI).

Concerning the complexity of the subjunctive, some researchers [80,122,123] argue that the late acquisition of the subjunctive in French L2 is due to the few occurrences of the subjunctive in native French speech. For example, Laurier [80], for Canadian French, found only 283 out of 989 contexts where the subjunctive was produced in 117 interviews, each lasting half an hour. He also found that in 273 contexts, the subjunctive was used with high-frequency verbs, and 8 were optional contexts of use. On the contrary, Gudmestand and Edmonds [124], using two elicitation tasks, found 1640 occurrences of the subjunctive corresponding to 53.3% of the overall subjunctive and consequently in the learners’ input. Despite this argument, we have seen that French natives begin to use the subjunctive at the age of 2 [76] and that it is stabilized at the age of 7 (sometimes even at the age of 4, as in the research reported by Bassano [77]). It has been shown by Bartning and Schlyter (2004) that learners use the subjunctive at different stages of the learning process of French. Research attested these development stages by using tasks including oral productions and written texts of learners from different L1 backgrounds that were less or more typologically distant from French. Most of the studies ([107,125,126,127,128,129,130,131,132] among others) stress the importance of the linguistic context in L2 learning and the impact of it on language use and development. It is worth pointing out that there is no doubt that the context of learning an L2 (traditional classroom, immersion learning in a realistic language context, or a virtual context, such as a laboratory) performs a role in L2 acquisition, as it has effects on the L2 language production and development. Students who have spent time in countries where French is spoken have many more opportunities to use their L2 knowledge than students in their home country who cannot practice their French. This undoubtedly increases their knowledge of how to use the subjunctive.

Taken together, these studies suggest that the pathway of acquisition toward the subjunctive is the following: present > imperfect > future > conditional > subjunctive. According to Bybee [133,134], some verbal forms of flexional paradigms can be interpreted as the verb root form + flexional ending (e.g., manger > *je mange* ‘I eat’ (present indicative) > *je manger + ai* ‘I’ll eat’ (simple future). She states that when children and learners of an L2 acquire verbal conjugation they start by acquiring those verb forms that can be used to replace other forms. These verb forms are bases for deriving other verb forms, as shown in the example above. In most cases, the base form of the verb, in French and some other Romance languages, is the present singular of the indicative (e.g., Spanish and Italian). Her suggestion had already been echoed by Guillaume [135], who showed that among nursery school children, third conjugation verbs (*-re*/*-oir*) were used more often than the first conjugation (*-re*) in French. However, the verbs of the third conjugation, which were frequent, occurred in a small number of forms. On the other hand, the first conjunction occurred less frequently but represented a great proportion of verb forms ending in -*er* (*-er* = 124 verbs out of 1060 uses (76%) vs. verbs ending in—*re*/*oir* 29 verbs out of 1706 uses (17.9%), [135]. Wilmet [136] proposed to consider conditional and subjunctive moods as tenses of the indicative because both are created on verbal bases of the indicative or withinflexionall endings of indicative tenses.

These findings might help to explain why French L2 learners first acquire the imperfect, then the future, moving on then to the conditional, and last, to the subjunctive. Once they have acquired the verbal forms of the present tense of the indicative, they can replicate these forms for other tenses and modes, as in the case of the conditional and subjunctive. Conditional contexts appear before the subjunctive, maybe because future inflections are easier to apply to the verbal forms to conjugate the conditional than imperfect ones (for the subjunctive). Once learners have understood that they can just add an “s” to the inflexional endings of the future indicative, they can use the conditional. For the subjunctive, the matter is more complicated. On the one hand, verbs ending in -er (e.g., *manger* ‘to eat’) are more frequent than verbs ending in -*ir* (e.g., *rougir* ‘to blush’), or than verbs ending in -re/oir (*vendre* ‘to sell’/*concevoir* ‘to understand’). Additionally, for verbs ending in -er, there is no morphophonological difference between the indicative and the subjunctive; both are formally realized identically (indicative = *Je mange* ‘I eat’, subjunctive = *Il faut que je mange* ‘I have to eat’). It is likely that acquiring a linguistic phenomenon that is not morphophonologically salient might be difficult for learners of French. In addition, the subjunctive has two bases: one for the first three persons of the singular and the third person of the plural (*que je vienne* [vjɛn], *tu viennes* [vjɛn], *il/elle vienne* [vjɛn], *ils/elles viennent* [vjɛn] ‘that I come, you come, he/she comes, they come’) and 2 for the 1st and 2nd persons of the plural (*que nous venions* [vənj$\tilde{ɔ}$], *vous veniez* [vənje], ‘that we come, you come’). Understanding, acquiring, and processing this system might be difficult and complex. It might take much longer than for the conditional mood. With irregular verbs, the acquisition might prove even more difficult because of the irregularities.

In French L2, we can track what learners have written/said at a precise moment, but we do not know if they really acquired the subjunctive long before using it. To date, no study has investigated if there is a dissociation, i.e., a time lag, between the production and processing capacities of the subjunctive. It appears plausible that learners may acquire the subjunctive but do not produce it for some reasons, as suggested by VanPatten and Jereski (2010, p. 451) [137], who argue that the acquisition of features of grammatical knowledge “does not automatically entail the processing of something related”. We can infer that there might be a gap between cognitive and neurocognitive processing and the production of the subjunctive.

As for the *complexity* of processing in general, in the field of the neurocognition of language, at least two factors can be invoked: semantic and syntactic. Interestingly, the EEG technique of event-related brain potentials (ERP) allows us to differentiate language processes based on different neural responses. For example, it is now accepted in the literature on the cognitive neuroscience of language (for a review [57]) that semantic processes are rather related to a specific ERP component, i.e., the N400 [138,139], while syntactic processes are often related with an (E)LAN/P600 biphasic ERP complex. Critically the question of syntactic complexity pointed out in this review (see [52,53]) showed that syntactically correct sentences can modulate the amplitude of a frontal P600 as a function of their syntactic complexity. For the first time, these authors introduced an ERP component that differentiates *complexity* from *repair* and *revision* in syntactic analysis. Moreover, the benefit of being able to differentiate the different levels of linguistic analysis involved while processing the subjunctive, in our case, is crucial to decide which processing strategy (i.e., semantic or morphological) could be used by L2 learners of French when confronted with such syntactic complexity. This different scalp distribution might help to understand, in the case of L2 processing, if complex morphosyntactic structures are processed in the same way in natives and L2 learners. Moreover, quantitative/qualitative differences between native speakers and L2 learners for any language processes under investigation can be discussed by comparing both amplitude (in microvolts) and peak latency (in milliseconds) of specific ERPs. One could infer that linguistic complexity (higher demands in working memory and/or attentional resources) might have a different scalp distribution in L2 learners when it is rare/unpreferred and when it is expressed differently in learners’ L1. Additionally, the issue of the processing strategies used by L2 learners of French raises the question of the typological relationship between one’s L1 and L2 in determining how the underlying language processes work, in L2, depending on whether the syntactic structures involved are expressed differently or not in the learners’ L1 [96].

The fact remains that we still do not know if the subjunctive in French is neurocognitively processed in the same way by natives and learners, both quantitatively (amplitudes or latencies may differ in native speakers and French L2 learners) and qualitatively (different neurocognitive markers may be implicated). The existing literature has only investigated the production of the subjunctive, from a psycholinguistic point of view, in French-speaking communities. What is well established is that native-speaking French children acquire the subjunctive early in their language development. We can hypothesize that these “few occurrences”, as stated by some linguists, are sufficient for them to acquire the subjunctive but that they are not sufficiently frequent for learners of French L2. Maybe the problem is not so much the occurrences of the subjunctive but the fact that the neurocognitive processing of the subjunctive may differ in native French and French L2 learners. One could assume that, as remarked by some EEG and fMRI studies [9,140,141,142,143,144,145] (see [146] for a review), even if the same brain areas are recruited during L1 and L2 processing for syntactic structures, neurocognitive circuits are not the same for processing the L1 and the L2. Hence different brain neuronal correlates may be implicated in natives and L2 learners, at least at low levels of proficiency. For example, Jeon and Friederici [142] found that different brain correlates are recruited for L2 processing, namely the most anterior region of the prefrontal cortex, whereas for L1 processing, the posterior region is recruited. Interestingly, in an fMRI study, Luke and colleagues [143] found that English-Chinese bilinguals use different brain systems underlying Chinese reading during English sentence processing. These results might help us to explain why the subjunctive is difficult to acquire. If different neurocognitive markers at different levels of proficiency are implicated in the processing of the subjunctive by French L2 learners and French natives, one can infer that this discrepancy could complexify the task of acquiring and processing this verbal mood. If the subjunctive is processed differently from native speakers, as suggested by Clahsen and Felser [5,6,7,8] (e.g., by using semantic cues, and lexico-semantic driven processes), this would explain the difficulty of acquiring this mood and the complexity of processing it. 

EEG and fMRI studies demonstrated that with a high level of proficiency in L2, the same brain neuronal circuits are recruited during L1 and L2 processing. For example, Perani and colleagues [145] investigated the effect of the early and late acquisition of L2 in high and low-proficient bilinguals assessed with positron emission tomography (PET). Subjects were English-Italian bilinguals (aged between 19 to 50) who acquired English after the age of 10 years (high proficiency group, late acquisition bilinguals), Spanish-Catalan bilinguals who acquired their L2 before the age of 4 (high proficiency and early acquisition bilinguals), and a volunteer group of Italian native speakers of English, who learned English after the age of 10 at school (low proficiency group). Tasks consisted in listening to stories in their first language, in their second language, and in a completely foreign language (e.g., Japanese). Their results showed that the cortical responses of low-proficiency learners differed from those of natives. In natives and in high-proficiency learners, foci were observed in the left hemisphere in the temporal pole, the superior temporal sulcus, the middle temporal gyrus, and hippocampal structures. In contrast, for the low-proficiency learners they failed to find activation in the temporal poles or in the left anterior and posterior areas of the middle temporal gyrus.

Concerning the processing of the French subjunctive, further research should investigate the question of acquisition of mood first in French children, from a neurocognitive point of view, by using techniques, such as EEG or fMRI to better understand the neurocognitive processes implicated during the processing of this verbal mood and their precise location in the brain. The same paradigm should then be applied to analyze the processing of this mood in French adult native speakers in order to detect whether qualitative or quantitative differences or brain area activations, are present in native children and adults. According to SSH, there should be no difference in processing between children and adults; both should be capable of detecting morphosyntactic violations of the subjunctive. In contrast, the D/P model attributes a determining role to linguistic experience.

With respect to French L2 learners, given the two cognitive models of L2 syntactic processing, i.e., the SSH hypothesis and the D/P model, we strongly believe that the SSH hypothesis applies to our example of complexity in French. In fact, as shown by some EEG studies, when processing syntactically complex L2 structures, especially when they do not exist in the learners’ L1, learners process them by using semantic/pragmatic knowledge, at least transiently, at some stage of L2 learning. EEG/ERP allows us to study the use of such a processing strategy in L2 learners since there are well-identified neurophysiological markers associated with semantic and pragmatic processing (for a review, see [139]). Following the temporal dynamics of the stages of development of morphosyntax in late L2 learners proposed by Steinhauer et al. [67] and considering that the subjunctive is acquired only in later stages of language development [79,93,94,110,111] based on L1–L2 language distance/relation, one could predict the following ERP patterns (see Table 2) for the processing of the French subjunctive.

As displayed in Table 2 above, one can expect quantitative and qualitative differences in French L2 concerning L1–L2 typological relation/distance and proficiency levels. As the subjunctive is complex in French, one can predict that a small P600 ERP pattern will be found for low-proficiency Italian learners of French L2 and a biphasic LAN/P600 ERP pattern for high-proficiency Italian learners of French L2. This is because the subjunctive is expressed in Italian, so these learners should already have a cognitive form of this mood, even if functional contexts are different in French. For more distant L1–L2 learners, one can expect semantic processing, followed by a small P600 repair interpretation, in the early stages of acquisition. For high proficiency distant L1–L2 learners, one can predict two hypotheses: first, (1) a semantic followed by a syntactic process (N400 > P600) or (2) second, the emergence of a biphasic LAN/P600 pattern, quantitatively less pronounced than in natives.

To account for this hypothesis, different factors should be taken into consideration: time and the nature of exposure to the L2, type of L2 instruction, age of L2 acquisition, proficiency level (even in high proficiency levels, learners may have different levels based on the above-mentioned factors) (see Caffarra et al., 2019), linguistic dominance in L1 and L2 [147,148].

Thus, it could be interesting and fruitful, for future research, to examine the neurocognitive markers responsible for the processing of the subjunctive in French native speakers and French L2 learners. Those which merit attention are (E)LAN for morphosyntactic processes [54,55,56,57,64]; see [61] for a review), N400 is thought to reflect lexical-semantic integration [138], the posterior P600, which is known to be associated with repair and reanalysis [149], and the frontal P600, which is assumed to mark the analysis of syntactic complexity [53]. Further research should investigate its processing with neurocognitive techniques that might help us to understand the neurocognitive processes involved. Quantity and quality differences using EEG, for example, in French native speakers and French L2 could help us to better explore this grammatical complexity. Combined research from production and processing is a future path to follow in order to understand if learners of French L2 present a dissociation between their processing/comprehension capacities and their production capacities of this grammatical complexity.

To conclude this review, we have tried to show some directions to be followed to answer the research questions we proposed at the beginning. For the first question, current research indicates that complexity at different levels of linguistic analyses may involve different neurocognitive processing mechanisms in L1 and L2. These mechanisms may be expressed qualitatively (different neurocognitive markers in L1 and L2 processing) and/or quantitatively (different latencies and/or amplitudes in native speakers and in L2 learners). For the second question, the literature shows that proficiency performs a crucial role in attaining native-like processes. Furthermore, evidence indicates that low-proficiency learners have more difficulties producing some complex L2 structures, suggesting that a high level is needed for specific L2 syntactic structures. Finally, the typological relation between L1–L2 seems to have a great impact on the attainment of native-like neurocognitive processing. Research suggests that when linguistic structures are similar in L1 and L2, learners are sensitive to morphosyntactic violations, but when these differ, learners cannot detect them. It may be because they use different neurocognitive mechanisms or more lexico-semantic driven procedures. In terms of pedagogical implications, research suggests [150] that the teaching of complex morphosyntactic structures in L2 depends on different factors, such as the proficiency levels of the learners, the type of instruction (formal vs. informal), and learner motivation. A contrastive L1–L2 approach could be useful for better understanding not only the forms the subjunctive is expressed by but also the modalities expressed by this verbal mood, particularly so for L1–L2 that are distant and where the subjunctive is expressed differently (e.g., Chinese and Japanese).

## Figures and Tables

**Table 1:** Contexts of subjunctive production in Howard’s study. Adapted from Howard [106].

	General Contexts	Contexts Identified in the Subjunctive with Irregular Verbs	Contexts Identified in the Subjunctive with both Regular and Irregular Verbs
Group 1	28	1	3
Group 2	26	3	9
Group 3	62	5	20

**Table 2:** The French subjunctive in French L2 acquisition: a hypothesis based on proficiency and on L1–L2 distance/relation.

−Distance/Relation L1–L2 (e.g., Italian and Spanish)		+Distance/Relation L1–L2 (e.g., Chinese and Japanese)	
Low Proficiency	High Proficiency	Low Proficiency	High Proficiency
quantitative small P600 ERP pattern	a biphasic LAN/P600 ERP pattern, quantitative differences may appear (amplitude and/or latency) w.r.t. natives.	N400 ERP pattern followed by a P600	1. Lexical processing N400, followed by a P600. 2. Emergence of LAN/P600 ERP pattern, quantitatively smaller than natives.

## Data Availability

Not applicable.
